# Supplementary material for: Estimation of Linkage Disequilibrium and Effective Population Size in Three Italian Autochthonous Beef Breeds
Source: Animals (Basel). 2020 Jun 14;10(6):1034. doi: 10.3390/ani10061034 (PMC7341513; doi:10.3390/ani10061034)
Supplement: Supplementary file 1 [file animals-10-01034-s001.zip › Supplem_table1.docx]

| Parameter^1^ | **CAL^2^** | **MUP** | **PON** | **LIM** |
| --- | --- | --- | --- | --- |
| nInd | 164 | 263 | 41 | 100 |
| rank | 163 | 262 | 40 | 99 |
| meanDiagonal | 0.99 | 0.99 | 0.97 | 0.99 |
| absmeanDiagonal | 0.99 | 0.99 | 0.97 | 0.99 |
| sqrmeanDiagonal | 0.99 | 0.99 | 0.95 | 0.98 |
| meanOffDiagonal | -0.006 | -0.004 | -0.024 | -0.010 |
| absmeanOffDiagonal | 0.06 | 0.10 | 0.09 | 0.02 |
| sqrmeanOffDiagonal | 3.72 | 1.45 | 0 | 0 |
| minDiag | 0.74 | 0.68 | 0.71 | 0.78 |
| maxDiag | 1.55 | 1.75 | 1.72 | 1.22 |
| minOffDiag | -0.16 | -0.21 | -0.20 | -0.08 |
| maxOffDiag | 0.79 | 1.10 | 0.84 | 0.50 |
| absminDiag | 0.74 | 0.68 | 0.71 | 0.78 |
| absmaxDiag | 1.55 | 1.76 | 1.72 | 1.22 |
| absminOffDiag | 3.47 | 1.52 | 0.00 | 0.97 |
| absmaxOffDiag | 0.79 | 1.1 | 0.84 | 0.50 |
| sqrminDiag | 0.54 | 0.46 | 0.5 | 0.61 |
| sqrmaxDiag | 2.40 | 3.08 | 2.97 | 1.50 |
| sqrminOffDiag | 0.02 | 0.05 | 0.04 | 0.01 |
| sqrmaxOffDiag | 0.63 | 1.20 | 0.70 | 0.25 |

**Table S1**: Genomic relationship matrix (GRM) summary statistics for each breed.

^1^nInd = number of individuals; rank = rank of the matrix; meanDiagonal = mean of diagonal; absmeanDiagonal = absolute value of the mean of diagonal; sqrmeanDiagonal = square root of the mean of diagonal; meanOffDiagonal = mean of off diagonal; asbmeanOffDiagonal = absolute value of the mean of off diagonal; sqrmeanOffDiagonal = square root of the mean of off diagonal; minDiag = minimum value of diagonal; maxDiag = maximum value of diagonal; minOffDiag = minimum value of off diagonal; maxOffDiag = maximum value of off diagonal; absminDiag = absolute value of the minimum value of diagonal; absmaxDiag = absolute value of the maximum value of diagonal; absminOffDiagonal = absolute value of the minimum value of off diagonal; absmaxOffDiag = absolute value of maximum value of off diagonal; sqrminDiag = square root of the minimum value of diagonal; sqrmaxDiag = square root of the maximum value of diagonal; sqrminOffDiag = square root of the minimum value of off diagonal; sqrmaxOffDiag = square root of the maximum value of off diagonal.

^2^CAL = Calvana; MUP = Mucca Pisana; PON = Pontremolese; LIM = Limousine.
