# Supplementary material for: Estimation of Linkage Disequilibrium and Effective Population Size in Three Italian Autochthonous Beef Breeds
Source: Animals (Basel). 2020 Jun 14;10(6):1034. doi: 10.3390/ani10061034 (PMC7341513; doi:10.3390/ani10061034)
Supplement: Supplementary file 1 [file animals-10-01034-s001.zip › Supplem_table2.docx]

| **CAL^1^** | | | | | | | **MUP** | | | | | | **PON** | | | | | **LIM** | | | | | |
| --- | --- | --- | --- | --- | --- | --- | --- | --- | --- | --- | --- | --- | --- | --- | --- | --- | --- | --- | --- | --- | --- | --- | --- |
| Autosome | N° SNP | Length ^2^ (Mbp) | Mean distance^3^ (Mbp) | SD distance^4^ (Mbp) | Longest interval ^5^ (Mbp) | N° SNP | | Length (Mbp) | Mean distance (Mbp) | SD distance (Mbp) | Longest interval (Mbp) | N° SNP | | Length (Mbp) | Mean distance (Mbp) | SD distance (Mbp) | Longest interval (Mbp) | | N° SNP | Length (Mbp) | Mean distance (Mbp) | SD distance (Mbp) | Longest interval (Mbp) |
| 1 | 1328 | 158.86 | 0.12 | 0.09 | 0.54 | 1304 | | 158.86 | 0.12 | 0.10 | 0.59 | 1282 | | 158.32 | 0.12 | 0.10 | 0.64 | | 1324 | 158.86 | 0.12 | 0.09 | 0.62 |
| 2 | 1163 | 136.67 | 0.12 | 0.09 | 0.60 | 1080 | | 136.68 | 0.13 | 0.10 | 0.74 | 1073 | | 136.48 | 0.13 | 0.10 | 0.74 | | 1079 | 136.67 | 0.13 | 0.10 | 0.57 |
| 3 | 1080 | 121.37 | 0.11 | 0.09 | 0.57 | 1070 | | 121.38 | 0.11 | 0.09 | 0.63 | 1039 | | 121.28 | 0.12 | 0.09 | 0.57 | | 1086 | 121.37 | 0.11 | 0.09 | 0.57 |
| 4 | 1013 | 120.53 | 0.12 | 0.08 | 0.53 | 1030 | | 120.62 | 0.12 | 0.08 | 0.53 | 984 | | 120.53 | 0.12 | 0.09 | 0.55 | | 1011 | 120.62 | 0.12 | 0.08 | 0.53 |
| 5 | 1205 | 121.08 | 0.10 | 0.08 | 0.74 | 1209 | | 121.08 | 0.10 | 0.08 | 0.74 | 1176 | | 120.90 | 0.10 | 0.09 | 0.74 | | 1222 | 121.08 | 0.10 | 0.08 | 0.60 |
| 6 | 1093 | 121.36 | 0.11 | 0.10 | 1.96 | 1144 | | 121.36 | 0.11 | 0.10 | 1.96 | 1109 | | 119.34 | 0.11 | 0.10 | 1.96 | | 1143 | 121.36 | 0.11 | 0.10 | 1.96 |
| 7 | 969 | 112.61 | 0.12 | 0.09 | 1.18 | 945 | | 112.61 | 0.12 | 0.10 | 1.18 | 927 | | 112.55 | 0.12 | 0.10 | 1.18 | | 929 | 112.61 | 0.12 | 0.10 | 1.18 |
| 8 | 909 | 113.32 | 0.13 | 0.09 | 0.71 | 885 | | 113.32 | 0.13 | 0.09 | 0.71 | 859 | | 113.30 | 0.13 | 0.10 | 0.71 | | 871 | 113.32 | 0.13 | 0.09 | 0.54 |
| 9 | 919 | 105.64 | 0.12 | 0.09 | 0.64 | 898 | | 105.64 | 0.12 | 0.09 | 0.51 | 887 | | 105.55 | 0.12 | 0.09 | 0.87 | | 898 | 105.64 | 0.12 | 0.09 | 0.51 |
| 10 | 917 | 104.22 | 0.11 | 0.08 | 0.90 | 915 | | 104.22 | 0.11 | 0.07 | 0.53 | 878 | | 104.19 | 0.12 | 0.08 | 0.61 | | 89 | 104.22 | 0.12 | 0.08 | 0.89 |
| 11 | 1016 | 107.25 | 0.11 | 0.08 | 0.93 | 974 | | 107.25 | 0.11 | 0.08 | 0.93 | 958 | | 107.24 | 0.11 | 0.08 | 0.78 | | 981 | 107.25 | 0.11 | 0.08 | 0.93 |
| 12 | 778 | 91.09 | 0.12 | 0.14 | 2.90 | 742 | | 91.09 | 0.12 | 0.14 | 2.87 | 738 | | 91.06 | 0.12 | 0.12 | 2.09 | | 742 | 91.09 | 0.12 | 0.18 | 4.44 |
| 13 | 731 | 84.08 | 0.12 | 0.09 | 0.86 | 736 | | 84.15 | 0.11 | 0.09 | 0.75 | 707 | | 84.08 | 0.12 | 0.09 | 0.75 | | 711 | 84.15 | 0.12 | 0.10 | 1.13 |
| 14 | 835 | 84.62 | 0.10 | 0.08 | 0.51 | 829 | | 84.62 | 0.10 | 0.08 | 0.61 | 773 | | 84.59 | 0.11 | 0.09 | 0.58 | | 813 | 84.62 | 0.10 | 0.08 | 0.58 |
| 15 | 814 | 85.22 | 0.11 | 0.08 | 0.47 | 798 | | 85.22 | 0.11 | 0.09 | 0.60 | 779 | | 85.14 | 0.11 | 0.09 | 0.60 | | 793 | 85.22 | 0.11 | 0.09 | 0.60 |
| 16 | 728 | 81.54 | 0.11 | 0.09 | 0.61 | 737 | | 81.54 | 0.11 | 0.09 | 0.74 | 695 | | 81.45 | 0.12 | 0.10 | 0.74 | | 714 | 81.54 | 0.11 | 0.10 | 1.03 |
| 17 | 709 | 74.97 | 0.11 | 0.08 | 0.80 | 697 | | 74.97 | 0.11 | 0.08 | 0.80 | 678 | | 74.95 | 0.11 | 0.08 | 0.80 | | 695 | 74.97 | 0.11 | 0.07 | 0.77 |
| 18 | 796 | 65.98 | 0.08 | 0.07 | 0.37 | 788 | | 65.98 | 0.08 | 0.07 | 0.46 | 791 | | 65.84 | 0.08 | 0.07 | 0.54 | | 803 | 65.98 | 0.08 | 0.07 | 0.46 |
| 19 | 781 | 64.01 | 0.08 | 0.07 | 0.47 | 793 | | 64.01 | 0.08 | 0.07 | 0.75 | 783 | | 63.98 | 0.08 | 0.07 | 0.55 | | 791 | 64.01 | 0.08 | 0.07 | 0.55 |
| 20 | 782 | 71.96 | 0.09 | 0.08 | 0.51 | 817 | | 71.96 | 0.09 | 0.08 | 0.47 | 774 | | 71.90 | 0.09 | 0.09 | 0.65 | | 792 | 71.96 | 0.09 | 0.08 | 0.61 |
| 21 | 704 | 71.51 | 0.10 | 0.08 | 0.41 | 698 | | 71.51 | 0.10 | 0.10 | 1.52 | 680 | | 71.41 | 0.10 | 0.10 | 0.98 | | 693 | 71.51 | 0.10 | 0.10 | 1.57 |
| 22 | 606 | 61.38 | 0.10 | 0.07 | 0.49 | 607 | | 61.38 | 0.10 | 0.07 | 0.43 | 572 | | 61.33 | 0.11 | 0.07 | 0.42 | | 596 | 61.38 | 0.10 | 0.07 | 0.39 |
| 23 | 626 | 52.47 | 0.08 | 0.07 | 0.72 | 634 | | 52.47 | 0.08 | 0.07 | 0.78 | 610 | | 52.41 | 0.09 | 0.08 | 0.78 | | 613 | 52.47 | 0.09 | 0.08 | 0.85 |
| 24 | 615 | 62.64 | 0.10 | 0.07 | 0.48 | 588 | | 62.64 | 0.11 | 0.08 | 0.70 | 572 | | 62.63 | 0.11 | 0.08 | 0.48 | | 577 | 62.64 | 0.11 | 0.08 | 0.48 |
| 25 | 516 | 42.85 | 0.08 | 0.06 | 0.38 | 528 | | 42.85 | 0.08 | 0.06 | 0.38 | 518 | | 42.77 | 0.08 | 0.06 | 0.38 | | 518 | 42.77 | 0.08 | 0.06 | 0.38 |
| 26 | 523 | 51.68 | 0.10 | 0.07 | 0.48 | 518 | | 51.68 | 0.10 | 0.07 | 0.41 | 509 | | 51.64 | 0.10 | 0.07 | 0.44 | | 526 | 51.68 | 0.10 | 0.07 | 0.41 |
| 27 | 451 | 45.37 | 0.10 | 0.08 | 0.85 | 442 | | 45.37 | 0.10 | 0.08 | 0.85 | 435 | | 45.35 | 0.10 | 0.08 | 0.85 | | 443 | 45.37 | 0.10 | 0.08 | 0.96 |
| 28 | 486 | 46.25 | 0.10 | 0.06 | 0.34 | 483 | | 46.25 | 0.10 | 0.06 | 0.48 | 469 | | 46.07 | 0.10 | 0.06 | 0.39 | | 479 | 46.19 | 0.10 | 0.06 | 0.56 |
| 29 | 552 | 51.50 | 0.09 | 0.07 | 0.52 | 547 | | 51.50 | 0.09 | 0.07 | 0.51 | 535 | | 51.47 | 0.10 | 0.07 | 0.50 | | 545 | 51.50 | 0.09 | 0.07 | 0.51 |

**Table S2**: Summary of analyzed SNPs for each breed, split by autosomal chromosomes.

^1^CAL = Calvana; MUP = Mucca Pisana; PON = Pontremolese; LIM = Limousine; ^2^ autosome length; ^3^ mean distance between two pairs of SNPs; ^4^ standard deviation of the distance between two pairs of SNPs; ^5^ the longest interval between two pairs of SNPs.
