# Supplementary material for: Estimation of Linkage Disequilibrium and Effective Population Size in Three Italian Autochthonous Beef Breeds
Source: Animals (Basel). 2020 Jun 14;10(6):1034. doi: 10.3390/ani10061034 (PMC7341513; doi:10.3390/ani10061034)
Supplement: Supplementary file 1 [file animals-10-01034-s001.zip › Suppl_Table3.docx]

***Table S3.*** Average and standard deviation (SD) of linkage disequilibrium (*r^2^*), estimated in 4 intervals of 0.25 Mbp, per breed.

| **Breed^1^** | **CAL** | | **MUP** | | **PON** | | **LIM** | | |
| --- | --- | --- | --- | --- | --- | --- | --- | --- | --- |
| **Distance range (Mbp)** | **Average *r^2^*** | **SD** | **Average *r^2^*** | SD | Average *r^2^* | SD | | Average *r^2^* | SD |
| 0 - 0.25 | 0.194 | 0.256 | 0.190 | 0.249 | 0.223 | 0.271 | | 0.141 | 0.224 |
| 0.25 - 0.5 | 0.130 | 0.177 | 0.121 | 0.161 | 0.155 | 0.193 | | 0.050 | 0.074 |
| 0.5 - 0.75 | 0.118 | 0.161 | 0.113 | 0.152 | 0.149 | 0.184 | | 0.040 | 0.058 |
| 0.75 - 1 | 0.110 | 0.151 | 0.108 | 0.146 | 0.145 | 0.179 | | 0.035 | 0.052 |

^1^ CAL = Calvana; MUP = Mucca Pisana; PON = Pontremolese; LIM = Limousin.
