# Supplementary figures and images for: Estimation of Linkage Disequilibrium and Effective Population Size in Three Italian Autochthonous Beef Breeds
Source: Animals (Basel). 2020 Jun 14;10(6):1034. doi: 10.3390/ani10061034 (PMC7341513; doi:10.3390/ani10061034)

CAL

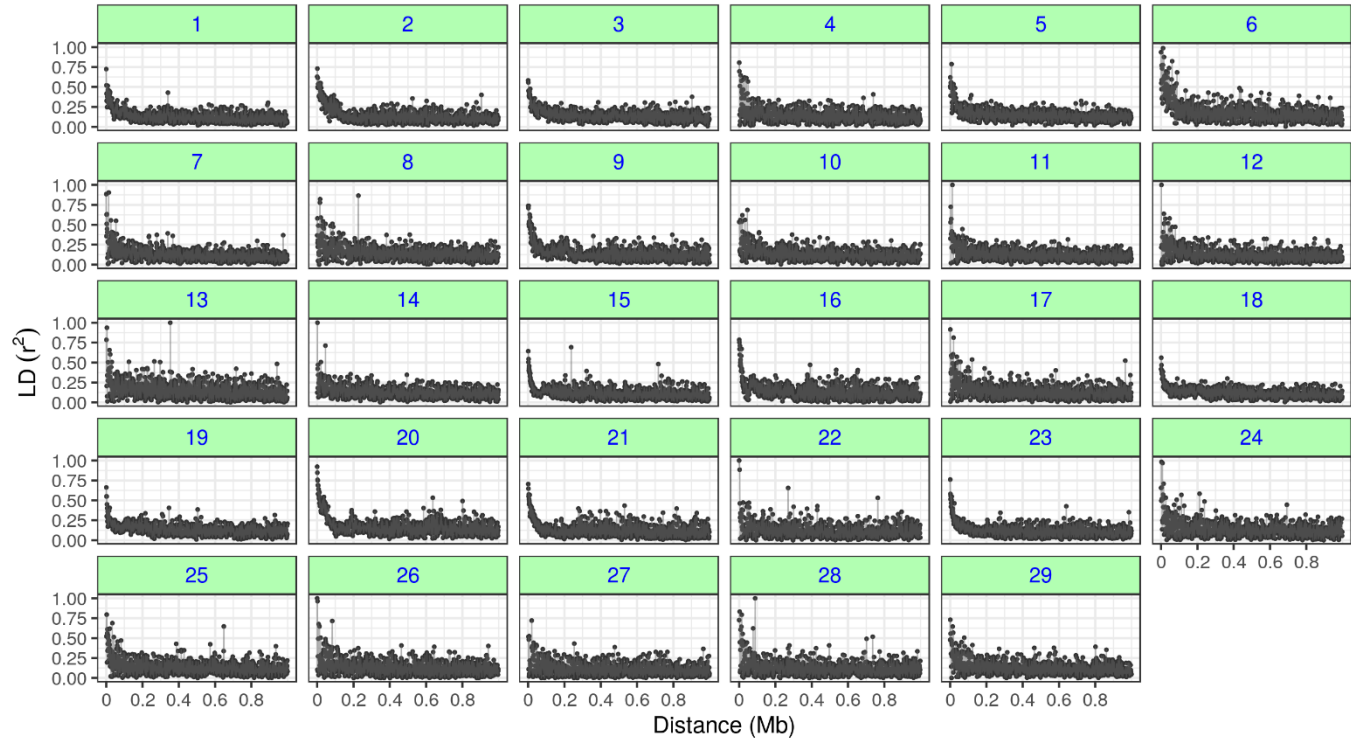

MUP

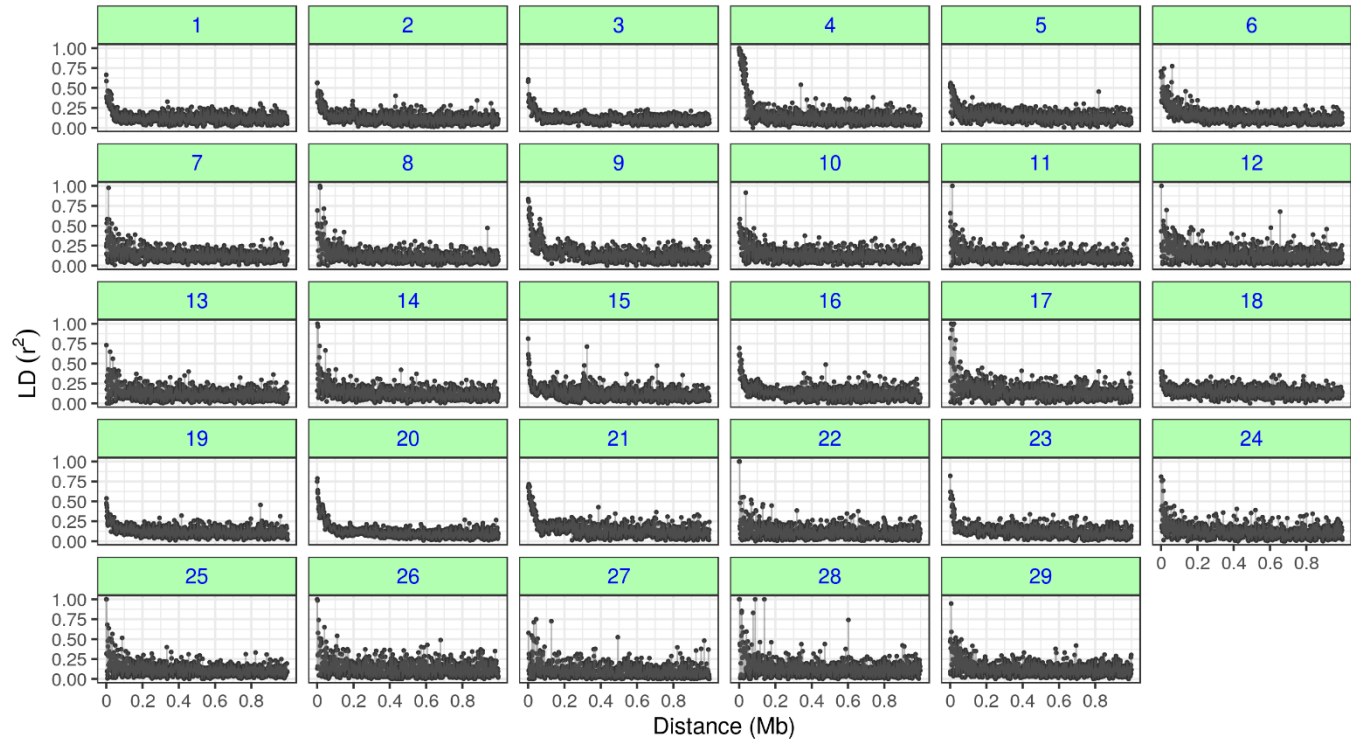

PON

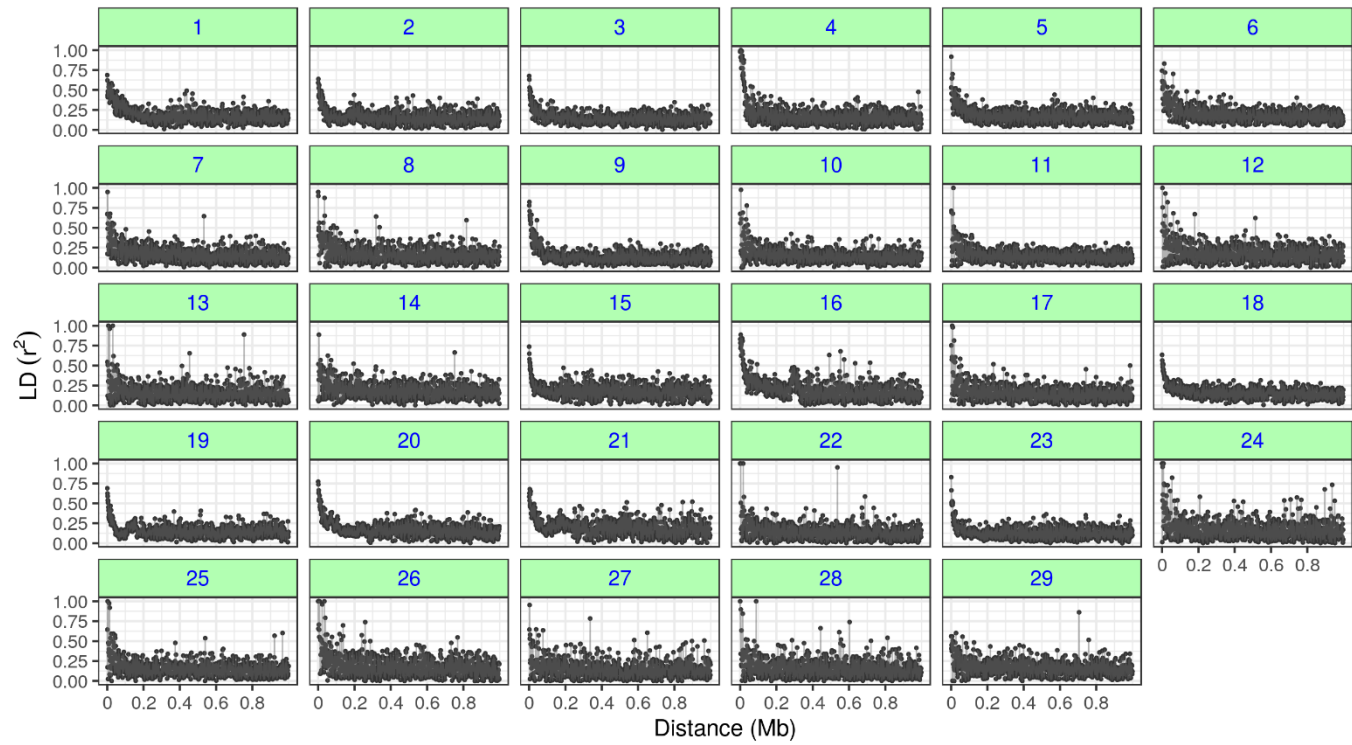

LIM

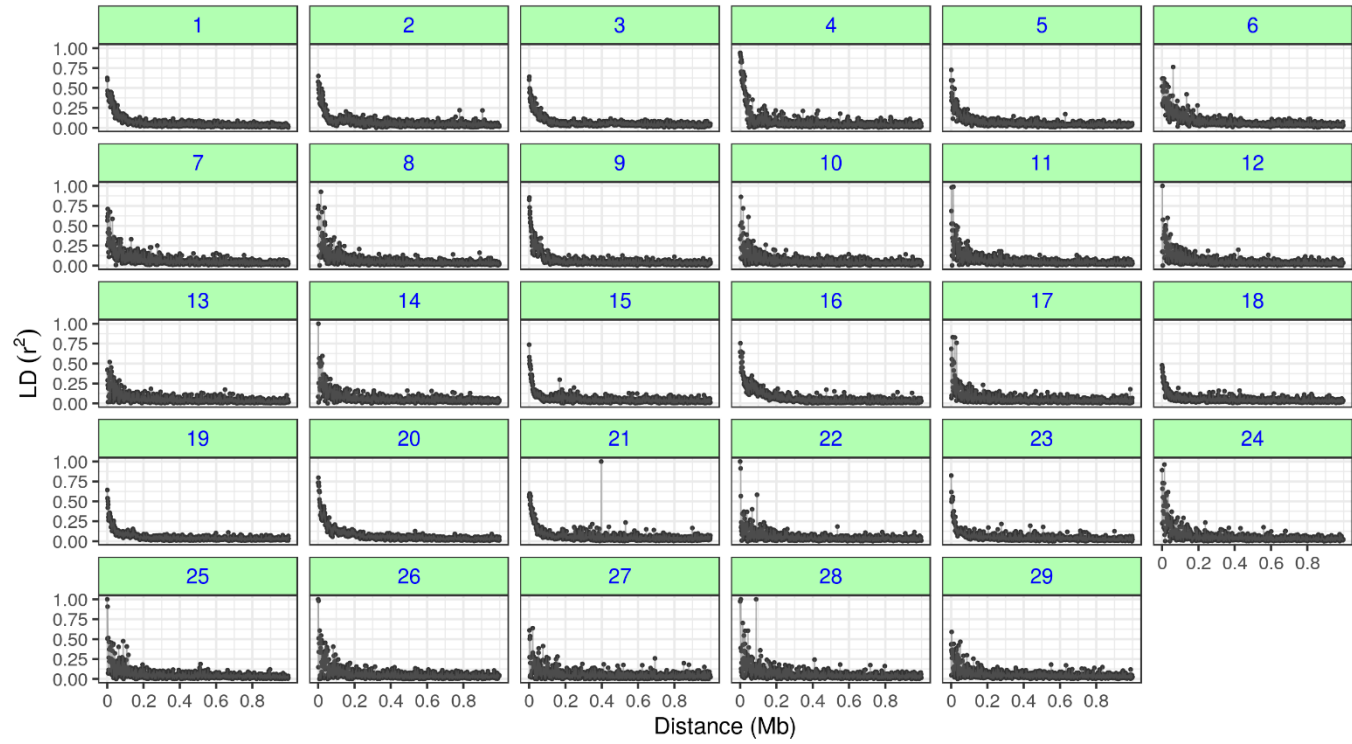

Supplement: Supplementary file 1 [file animals-10-01034-s001.zip › Suppl_Figure1.pdf]
